# A Retrospective Analysis of Liberal Fluid Therapy and Total Protein Changes in 124 Horses Undergoing Colic Surgery

**DOI:** 10.3390/ani16192998

**Published:** 2026-09-23

**Authors:** Marco Gandini, Beatrice Dal Cero, Paolo Franci, Gessica Giusto

**Affiliations:** 1 Department of Veterinary Sciences, University of Turin, Largo Paolo Braccini 2–5, 10095 Grugliasco, TO, Italy; paolo.franci@unito.it (P.F.); gessica.giusto@unito.it (G.G.); 2 Equine Education and Clinical Department, University of Liège, Vallée 2 District, 5 Avenue de Cureghem, 4000 Liège, Belgium; dalcerobeatrice@gmail.com

**Keywords:** colic surgery, liberal fluid therapy, haemodilution, hypoproteinaemia, overhydration, total plasma protein, fluid responsiveness

## Abstract

Perioperative fluid therapy in horses undergoing colic surgery should be individualised to avoid complications associated with both hypo- and hypervolaemia. This retrospective study evaluated the haemodynamic effects of a liberal perioperative fluid therapy approach, focusing on total plasma protein (TPP) changes. Medical records of 124 horses undergoing colic surgery between March 2018 and June 2020 were reviewed. Packed cell volume (PCV) and TPP were assessed preoperatively, after surgery, on postoperative days (POD) 2, 5 and 7, and at long-term follow-up. Horses were stratified according to postoperative TPP (≤5 vs. >5 g/dL) and lesion type (strangulating vs. non-strangulating). All horses received crystalloids (median 34.3 mL/kg; range 9–99 mL/kg), while 41/124 also received colloids (HES 130/0.4, 4 mL/kg). TPP was significantly lower at anaesthetic recovery than at admission at the population level (*p* < 0.0001). At the individual level, recovery TPP was lower than POD7 in 106/124 horses (85.5%), while 52/124 horses (41.9%) had a recovery TPP concentration ≤ 5 g/dL. Values progressively returned toward baseline from POD2 onwards. In non-strangulating lesions, horses with TPP ≤ 5 g/dL received substantially greater crystalloid volumes than horses with TPP > 5 g/dL (56.8 vs. 31.5 mL/kg), whereas PCV changes were comparable between groups. The association between crystalloid dose and TPP reduction remained significant after adjustment for lesion type and intestinal tract involved; however, residual confounding by unmeasured indicators of disease severity cannot be excluded. Greater cumulative crystalloid administration was associated with a greater magnitude of TPP reduction. Horses with post-recovery TPP ≤ 5 g/dL had a significantly longer hospital stay than those with TPP > 5 g/dL (*p* = 0.006). Serial TPP monitoring may provide a practical, real-time measure of changes in plasma protein concentration and perioperative haemodilution; its role in guiding fluid administration requires prospective validation.

## Simple Summary

Horses undergoing surgery for colic often require large amounts of intravenous fluids to maintain blood circulation and blood pressure during anaesthesia and recovery. However, excessive fluid administration may dilute blood proteins and contribute to postoperative complications. This study investigated how perioperative fluid therapy affected blood protein levels in 124 horses undergoing colic surgery. Blood samples collected before and after surgery showed that protein levels decreased significantly in many horses after anaesthesia, particularly in horses receiving larger volumes of fluids. Although TPP decreased perioperatively in many horses, 52/124 horses (41.9%) had a TPP concentration ≤ 5.0 g/dL at anaesthetic recovery. In contrast, changes in red blood cell concentration were less useful for identifying fluid overload. These findings show an association between the amount of crystalloid administered and the magnitude of the postoperative reduction in blood protein concentration. Monitoring blood protein concentrations during surgery may therefore provide veterinarians with a simple additional measure of haemodilution during fluid administration. However, this study did not directly assess circulating blood volume, fluid responsiveness, or the effects of haemodilution on postoperative complications, and the potential role of protein monitoring in guiding fluid therapy requires prospective investigation.

## 1. Introduction

Perioperative fluid management is a therapeutic cornerstone in patients undergoing gastrointestinal surgery, in both human and veterinary medicine [1,2]. In human surgery, patients undergoing gastrointestinal procedures are particularly prone to develop deleterious consequences on recovery of gastrointestinal motility, wound and anastomotic healing, coagulation, and cardiopulmonary function when subjected to overzealous fluid administration [1,2,3,4,5]. While the choice of fluid type and volume remains subject to vigorous debate, the general principle of shifting towards an individualised fluid strategy is widely accepted across species [3].

Colic horses present a wide range of clinical states, from mild dehydration to hypovolaemic shock. The use of fluid therapy unrelated to the patient’s actual needs can be detrimental for both hypo- and hyper-volaemic horses, and several authors have highlighted the need for an individualised perioperative fluid strategy [6,7,8,9,10,11]. However, matching perioperative fluid requirements in horses undergoing colic surgery is a challenging clinical task, also because some of the most commonly used haemodynamic point-of-care parameters—PCV, TPP—have low specificity and sensitivity for detecting true hypo- or hypervolaemia and fluid responsiveness. PCV is also strongly influenced by splenic contraction, while TPP may be altered by specific disease processes (e.g., protein leakage in large colon torsion) or by endotoxaemia-associated increases in acute-phase proteins [12,13]. In an emergency setting, a ‘fluid challenge’ [9] is not always feasible in a timely manner.

Clinicians typically follow general guidelines, which in horses with colic suggest an infusion rate of 3–10 mL/kg/h during anaesthesia, with a stated tolerance of up to 45 mL/kg/h in adult horses [10,11] and about 80 mL/kg/h in healthy horses [9]. To replace losses and counteract the effects of shock, administration of boluses of a total rate ranging from 20 to 90 (‘shock dose’) mL/kg of crystalloids is described [12,13,14]. The effects of this wide-boundary, effectively liberal approach on blood parameters have never been systematically evaluated in a large population of colic horses, though raising clinical and scientific interest in different contexts [15].

Despite some previous awareness of these issues [16,17,18,19], only recently has systematic attention been given to individualising fluid administration to patient needs [6,20,21]. The adverse effects of large-volume fluid administration—particularly haemodilution-induced hypoproteinaemia—have been largely overlooked [21]. Rapid fluid bolus administration, often used to stabilise critical colics, is associated with adverse effects on the microcirculation and the endothelial surface layer (ESL), the protective barrier, including the glycocalyx, which regulates transvascular fluid movement, vasomotor tone, coagulation, and inflammation [22]. Thinning or shedding of this critically important layer through rapid intravascular volume expansion may promote interstitial oedema. Direct evidence of endothelial glycocalyx damage has recently been reported in horses with intestinal disease: in a study of 61 horses (healthy controls, non-strangulating inflammatory disease, and strangulating lesions), plasma and peritoneal fluid concentrations of glycocalyx components (syndecan-1, heparan sulfate, hyaluronan) were elevated in horses with intestinal disease, particularly strangulating lesions, and were higher in non-survivors than in survivors [23]. With the size of equine patients and the need for large and rapid volumes of fluid to correct deficits, this presents difficulties with respect to preservation of the ESL. Fluid overload can cause intestinal oedema, damage the endothelial glycocalyx, dilute coagulation factors, and ultimately worsen outcome, as demonstrated in small animal medicine, in experimental models, and increasingly in horses [24,25].

The primary objective of the present study was to quantify the association between cumulative perioperative crystalloid administration and the magnitude of TPP reduction in horses undergoing colic surgery. Secondary objectives were to describe perioperative changes in TPP and PCV, to evaluate whether the association between crystalloid administration and TPP reduction persisted after adjustment for the available disease-related variables, and to explore the potential utility of TPP as a practical point-of-care marker of perioperative haemodilution.

## 2. Materials and Methods

### 2.1. Study Population and Inclusion Criteria

Retrospective data from horses submitted to colic surgery at the Veterinary Teaching Hospital (VTH) of Turin between March 2018 and June 2020 were retrieved. All horses included in the study received pre- and intraoperative fluids according to the current literature guidelines for perioperative fluid therapy in colic horses [10,11,12,13,14]. Because this approach leaves the clinician free to administer fluids within a wide dosing range, the current perioperative fluid therapy regime is referred to as ‘liberal’ throughout this article, used here strictly as a descriptive term for the wide, clinician-determined dosing range applied in our hospital, and not as an endorsement of high-volume fluid administration; indeed, the wide guideline ranges cited above are intended to allow restriction as much as expansion of fluid volume, and our findings argue in favour of titrating within this range rather than defaulting to its upper end.

Exclusion criteria were (1) death or euthanasia before discharge; (2) diseases potentially causing hypoproteinaemia independent of fluid therapy (e.g., strangulating lesions of the large colon, recognising that other diseases, particularly prolonged small intestinal strangulation, may also independently affect protein status, as discussed in the Limitations); and (3) administration of postoperative intravenous fluids. Since horses in our hospital are managed under a postoperative goal-directed fluid therapy (GDFT) protocol and are not routinely administered postoperative fluids, exclusion of those that did receive postoperative fluids allowed us to remove postoperative fluid therapy as a source of variation in perioperative protein and haematocrit dynamics, although pre- and intraoperative fluid administration itself was not standardised or randomised.

### 2.2. Data Collection

PCV and TPP values were obtained at the following time points: (1) arrival; (2) immediately before surgery; (3) after recovery from anaesthesia (hereafter ‘recovery’); and (4) POD 2, 5, and 7. Routine postoperative blood samples during hospitalisation were obtained between 08:00 and 09:00. A blood sample was additionally obtained at least two months after discharge to define the time point at which each horse returned to its individual baseline TPP.

Data on the type and total amount of intravenous fluids administered between arrival and recovery from anaesthesia were recorded for each horse. Fluids administered included isotonic crystalloids (Lactated Ringer’s solution, LRS, Galenica Senese Srl, Monteroni D’Arbia, Siena, Italy) and, in selected cases, iso oncotic synthetic colloids (hydroxyethyl starch 130/0.4, HES; Voluven (R), Fresenius Kabi Italia Srl, Verona, Italy). For the small intestinal (SI) pathology subgroup (n = 52), fluid data were further stratified by TPP outcome group (≤5 g/dL vs. >5 g/dL at end of surgery) and by lesion type (strangulating [S] vs. non-strangulating [NS]).

Samples were processed with a standardised protocol using microhematocrit for PCV determination and a calibrated refractometer for TPP measurement [26]. The samples obtained at long-term follow-up were processed immediately upon receipt (within 6 h of sampling).

### 2.3. Statistical Analysis

Sample size was calculated using a web-based calculator (clincalc.com/stats/samplesize.aspx) assuming a postoperative mean TPP of 5.5 g/dL and a normal TPP value of 6.0 ± 0.2 g/dL (mean ± SD), with alpha = 0.05 and power = 90%. This led to a minimum required sample size of 4 horses per group (8 in total). The final cohort (*n* = 124) therefore exceeded the minimum statistical requirement. Continuous quantitative variables were assessed for normality using the Shapiro–Wilk test. For non-normally distributed data, the non-parametric Friedman test was used; results are reported as median (95% CI or interquartile range). Statistical significance was set at *p* < 0.05. Following significant Friedman tests, pairwise post hoc comparisons were performed using Wilcoxon signed-rank tests with Holm–Bonferroni correction for multiple comparisons. Between-group comparisons (e.g., TPP outcome groups) were assessed using the Mann–Whitney U test, with rank–biserial correlation reported as a measure of effect size. The relationship between perioperative crystalloid dose (mL/kg) and the percentage reduction in TPP (ΔTPP%) was assessed using Spearman’s rank correlation, followed by univariable and multivariable linear regression adjusting for lesion type (strangulating vs. non-strangulating) and intestinal tract involved (small vs. large intestine), to evaluate whether the association between crystalloid dose and TPP reduction persisted after adjustment for the available disease-related variables. Lesion type and intestinal tract involved were the disease-related variables consistently available for the entire retrospective cohort; other potentially relevant severity indicators, including lactate concentration, blood pressure, and degree of shock, were not sufficiently complete for inclusion in the model. All statistical analyses were performed with GraphPad Prism v.9.0 and Jamovi (the Jamovi project, version 26.1).

## 3. Results

### 3.1. Study Population

Records were available and complete for 191 colic cases (80 with small intestinal pathologies, 111 with large intestinal pathologies). Of these, 169/191 recovered from anaesthesia (70 SI, 103 LI) and 153/169 were discharged (58 SI, 95 LI). Twenty cases were excluded because of strangulating large colon lesions potentially causing protein loss independently of fluid therapy. Of the remaining 133 cases, nine (5 SI, 4 LI) received postoperative fluid therapy and were excluded. A total of 124 horses were included in the final analysis: 32 with strangulating SI pathologies, 21 with non-strangulating SI pathologies, and 71 with non-strangulating large intestinal pathologies.

### 3.2. Perioperative Fluid Administration

Crystalloids (LRS) were administered to all 124 horses perioperatively, with a median dose of 34.3 mL/kg (range 9–99 mL/kg). Colloids (HES 130/0.4, 4 mL/kg) were administered to 41/124 horses (32 with SI pathologies, nine with LI pathologies). Fluid administration data stratified by TPP outcome group and lesion type for the SI subgroup are summarised in Table 1.

Within the SI subgroup, horses in the non-strangulating subgroup that ended surgery with TPP ≤ 5 g/dL (Subgroup NS TPP ≤ 5) received a median of 56.8 mL/kg LRS compared to 31.5 mL/kg in the NS TPP > 5 subgroup (*p* = 0.156). In strangulating horses, median crystalloid volumes were 35.3 mL/kg (S TPP ≤ 5) vs. 28.3 mL/kg (S TPP > 5; *p* = 0.491). Despite higher crystalloid volumes in hypoproteinaemic horses, PCV reduction was similar across subgroups (Table 2), highlighting the dissociation between PCV and TPP trends during fluid loading.

A Spearman correlation confirmed a significant positive association between crystalloid dose (mL/kg) and the percentage reduction in TPP across the full cohort (ρ = 0.45, 95% CI 0.25–0.55, *p* < 0.0001). This association remained statistically significant in multivariable linear regression after adjustment for lesion type (strangulating vs. non-strangulating) and intestinal tract involved (β = 0.30, *p* = 0.006) (Figure 1).

### 3.3. TPP and PCV Dynamics over Time

At the population level, TPP was significantly lower at anaesthetic recovery than at admission (*p* < 0.0001; Table 3). At the individual level, 106/124 horses (85.5%) had a lower TPP concentration at recovery than at POD7, whereas 18/124 horses (14.5%) had a recovery TPP concentration equal to or greater than their POD7 value. Separately, 52/124 horses (41.9%) had a recovery TPP concentration ≤ 5.0 g/dL.

TPP values progressively increased from POD 2 onward and were statistically indistinguishable from long-term follow-up values from POD 2 (Table 4; *p* > 0.05 for all comparisons between POD 2, 5, 7 and long-term).

PCV also decreased significantly from arrival to recovery (*p* < 0.0001; Table 5). However, unlike TPP, its perioperative trend was markedly variable across individuals. The PCV at arrival was within 140% of the long-term PCV in the vast majority of cases, suggesting that splenic contraction rather than true dehydration may be responsible for the elevated admission PCV commonly observed in colic horses. Long-term PCV was lower than admission values (*p* = 0.018; Table 6), but did not differ significantly across intermediate timepoints.

### 3.4. Additional Clinical Correlates and Sensitivity Analyses

Horses with post-recovery TPP ≤ 5 g/dL had a significantly longer hospital stay than those with TPP > 5 g/dL (median 11 vs. 10 days; Mann–Whitney *p* = 0.006).

Among horses coded in hospital records as having developed postoperative ileus (POI; 14 cases identified in the broader hospital registry underlying this study), none remained in the final analytic cohort: all 14 had either died/been euthanised before discharge or had received postoperative intravenous fluids, both of which are exclusion criteria of the present study (Section 2.1), since POI was itself a primary clinical indication for postoperative fluid therapy under our protocol.

Effect sizes for Table 1 subgroup comparisons were as follows: in non-strangulating small intestinal lesions, the median difference in crystalloid dose between the TPP ≤ 5 and TPP > 5 subgroups was 19.9 mL/kg (95% CI 2.3–42.1; Mann–Whitney *p* = 0.014; rank–biserial r = −0.67); in strangulating small intestinal lesions, the corresponding median difference was 14.7 mL/kg (95% CI −5.8–37.8; *p* = 0.080; r = −0.39).

## 4. Discussion

The principal finding of this study was a frequent perioperative reduction in TPP that was positively associated with cumulative crystalloid administration. At the individual level, recovery TPP was lower than POD7 TPP in 106/124 horses (85.5%). Separately, 52/124 horses (41.9%) had a recovery TPP concentration ≤ 5.0 g/dL. These represent two distinct observations: the former describes the proportion of horses with a lower TPP at recovery than at POD7, whereas the latter identifies horses reaching the predefined low-TPP threshold at recovery. TPP subsequently increased from POD2 onwards, a temporal pattern compatible with a transient dilutional component. However, the observational design does not allow the relative contributions of perioperative fluid administration, underlying disease, and other determinants of plasma protein concentration to be established. Crystalloid dose was positively associated with the magnitude of TPP reduction, and this association remained statistically significant after adjustment for lesion type and intestinal tract involved. These variables represent only selected disease-related characteristics, and residual confounding by shock severity, lactate concentration, blood pressure, or other clinical determinants of fluid administration cannot be excluded. The cumulative crystalloid volumes administered (median 34.3 mL/kg; range 9–99 mL/kg) were within ranges described for horses undergoing laparotomy [27], indicating that substantial TPP reductions may occur within currently used dosing ranges. The clinical significance of these reductions cannot be established from the present study. In a post hoc exploratory analysis, horses with recovery TPP ≤ 5.0 g/dL had a statistically longer hospitalisation than horses with TPP > 5.0 g/dL (median 11 vs. 10 days); however, the absolute difference was small, and no causal relationship between lower postoperative TPP and prolonged hospitalisation can be inferred. This concept is in accordance with research studies that suggest that restricting the volume of crystalloid may preserve the endothelial layer and improve outcomes based on animal experimental models and preliminary clinical trials in people [22].

A legitimate question arising from our monitoring approach is why we rely on TPP rather than serum albumin or direct measurement of colloid osmotic pressure (COP), the latter being the more physiologically relevant determinant of transcapillary fluid flux and oedema formation.

The rationale is primarily practical. COP measurement requires a membrane osmometer, an instrument that is rarely available in equine clinical practice, even at university hospitals. Serum albumin quantification requires laboratory spectrophotometry or dye-binding methods (e.g., bromocresol green assay), which are neither immediate nor point-of-care accessible. By contrast, TPP is measured within seconds using a portable, calibrated hand-held refractometer, universally available in equine practice and yielding results in real time during surgery.

From a physiological standpoint, albumin contributes approximately 75–80% of COP in healthy horses. In acute haemodilutional states induced by rapid crystalloid infusion, both albumin and globulin fractions are diluted in parallel, meaning that TPP changes closely track COP changes under these specific circumstances [18,28]. In our population, having excluded large colon torsion (a common equine pathology associated with selective protein leakage and disproportionate albumin loss, although other gastrointestinal diseases, particularly prolonged small intestinal strangulation, can also cause disease-related hypoproteinaemia), the haemodilutional pattern is expected to affect all protein fractions proportionally. Under these conditions, serial TPP measurements may provide a practical point-of-care marker of changes in plasma protein concentration and the degree of haemodilution. 

A potential limitation is that, in individual horses, albumin may not fall in strict proportion to total protein—for instance, when acute-phase globulins are elevated due to marked endotoxaemia, in which selective globulin increases may partially maintain TPP despite actual albumin and COP reduction. In such cases, COP may be better maintained than TPP alone would suggest. Nevertheless, in the absence of bedside albumin or COP measurement, TPP monitoring represents a practical and readily accessible point-of-care method for monitoring changes in plasma protein concentration during perioperative fluid administration.

This target is broadly consistent with concerns raised by Boscan and Steffey [18], who investigated plasma colloid osmotic pressure and total protein in horses during colic surgery. While a single TPP value cannot be treated as an absolute cutoff given albumin variability, it provides a useful clinical target. It should be emphasised that no outcome-based cutoff analysis (e.g., ROC curve) was performed in the present study to formally derive this threshold; a TPP target of ≥5.0 g/dL at the end of surgery is therefore proposed as a pragmatic clinical target rather than as an evidence-based cutoff derived from this dataset.

PCV is one of the traditionally used parameters to guide fluid therapy in colic horses, with elevated values at admission prompting more aggressive fluid loading. However, PCV trends in our population were highly variable and not directly proportional to the amount of fluids administered, as demonstrated by the similar PCV reductions in groups with markedly different crystalloid volumes (Table 2).

One explanation could be the unique haematological physiology of the horse. The equine spleen can store up to one-third of circulating red blood cells; endogenous adrenaline release triggered by pain, stress, excitement, transport, or anaesthetic induction causes rapid splenic contraction and can increase haematocrit by more than 50% above resting values [29,30]. In our cases, PCV at admission was frequently within 140% of the long-term PCV—a range that would be consistent with splenic contraction; however, splenic contraction was not directly measured (e.g., by ultrasonographic assessment of splenic volume) in this study, and other contributors to admission PCV, including true dehydration, cannot be excluded on the basis of this observational dataset alone. This raises the possibility that the elevated PCV commonly observed at admission may partly reflect splenic contraction rather than true dehydration alone, suggesting that fluid therapy decisions based solely on PCV correction could carry a risk of unnecessary overhydration; confirmation of this hypothesis would require direct measurement of splenic volume or hydration status.

Furthermore, in some pathologies (e.g., nephrosplenic entrapment), PCV may paradoxically increase during surgery as the causative pathology is resolved. In other cases, concurrent subclinical piroplasmosis or mild surgical haemorrhage may cause independent PCV reductions unrelated to volaemic status. These confounders make PCV an unreliable single parameter for titrating fluid therapy in colic surgery. TPP, while also imperfect and influenced by disease-related factors, is not directly affected by splenic erythrocyte mobilisation and therefore provides complementary information regarding changes in plasma protein concentration during fluid administration.

The concept of fluid responsiveness—i.e., the ability of a patient to mount an increase in cardiac output following a fluid challenge, which underpins the decision of whether to administer further fluids—has been extensively studied in human medicine [29] and has only recently begun to be explored in equine medicine.

Fluid responsiveness is defined as an increase in cardiac output or stroke volume following fluid administration. Because cardiac output, stroke volume, and dynamic indices of fluid responsiveness were not measured in the present study, fluid responsiveness cannot be inferred directly from changes in TPP or PCV. In horses with non-strangulating small-intestinal lesions, those with recovery TPP ≤ 5.0 g/dL received a median of 56.8 mL/kg LRS compared with 31.5 mL/kg in horses with TPP > 5.0 g/dL, while median PCV reductions were similar between groups. These observations demonstrate differences in the biochemical response to fluid administration but do not establish whether the additional fluid increased circulating blood volume or cardiac output. Accordingly, TPP should be regarded as a marker of plasma protein concentration and haemodilution rather than as a direct measure of fluid responsiveness or intravascular volume. Whether serial TPP monitoring can help distinguish effective volume replacement from progressive fluid accumulation is an important hypothesis for prospective investigation. These considerations are consistent with the revised Starling framework discussed by Muir et al. [11], according to which general anaesthesia may alter transvascular fluid distribution and promote interstitial fluid accumulation. Experimental work in healthy horses has also demonstrated relatively slow elimination of administered crystalloids [15]. Together, these observations provide a physiological rationale for investigating whether fluid requirements differ according to disease characteristics. However, the present study was not designed to determine optimal fluid volumes for individual lesion types, and such differences require prospective evaluation using direct haemodynamic endpoints.

In strangulating pathologies, the affected intestinal segments are surgically removed, eliminating the primary source of inflammation and fluid sequestration intraoperatively. In non-strangulating conditions, the pathological bowel is decompressed but remains in situ, and pre-existing mucosal inflammation and altered motility may render it more susceptible to the accumulation of interstitial fluid from overhydration. Experimental studies indicate that intestinal oedema can impair intestinal contractile function [30], providing a potential mechanism by which excessive fluid accumulation could contribute to postoperative gastrointestinal dysfunction. Similarly, rapid intravascular volume expansion has been implicated in endothelial glycocalyx alterations, while recent equine evidence demonstrates increased circulating and peritoneal glycocalyx components in horses with intestinal disease [23]. However, intestinal oedema, glycocalyx integrity, postoperative ileus, and postoperative reflux were not evaluated as outcomes in the present study. These mechanisms should therefore be regarded as biological considerations supporting further investigation rather than as consequences demonstrated by our data. Prospective studies are required to determine whether optimal fluid requirements differ according to lesion type and disease severity.

An important methodological strength of this study—and a consideration with broader clinical implications—is that postoperative management at our VTH follows a standardised GDFT protocol, under which horses are not routinely administered postoperative intravenous fluids. Horses that did receive postoperative fluids (primarily for markedly elevated creatine kinase values or POI) were explicitly excluded from the analysis. This approach effectively eliminates postoperative fluid therapy as a confounding variable on TPP and PCV dynamics during hospitalisation. It should be noted that this standardisation was applied specifically to the postoperative period; intraoperative fluid administration itself was not standardised and was determined by the attending anaesthetist. A further methodological consequence of this exclusion criterion is that postoperative ileus (POI) could not be directly tested against TPP in the present dataset, since all identified POI cases fell outside the analytic cohort by design (Section 3.4).

Under this standardised postoperative protocol, analgesic agents (flunixin meglumine, butorphanol), gastroprotective medications (omeprazole), and antimicrobial regimens are applied uniformly. Enteral feeding is reintroduced according to standardised criteria, and vital parameters are monitored by the same clinical team. As a result, the number of variables that could independently influence early postoperative TPP trends is substantially reduced. The standardised postoperative protocol reduced variation attributable to postoperative intravenous fluid administration. However, it does not permit the early reduction in TPP to be attributed exclusively to perioperative fluid administration, because underlying disease, vascular permeability, protein loss, haemorrhage, and other unmeasured factors may also have contributed. 

This standardised approach also implies that, once perioperative fluid therapy is optimised, there are relatively few remaining modifiable variables within the postoperative period that could significantly alter early protein recovery trajectories. The standardised postoperative management used in this cohort reduced one potential source of variability in early postoperative TPP trajectories. Nevertheless, the observational design does not permit the relative contribution of perioperative fluid administration and other disease-related factors to be determined causally. Serial perioperative TPP monitoring may provide a simple and inexpensive means of identifying substantial changes in plasma protein concentration, while its potential role in guiding fluid administration and improving clinical outcomes requires prospective evaluation.

This study has several limitations. It is retrospective and non-randomised. Small and large intestinal pathologies were analysed together for the primary TPP/PCV trend analysis, which may introduce heterogeneity, although large colon torsion cases were excluded. PCV and TPP values were obtained by multiple operators, although procedures were standardised [26]. Values were measured with a microhematocrit and a refractometer; laboratory instruments might yield slightly different results. Although a significant association between crystalloid dose and TPP reduction was observed, contributions from intra-abdominal lavage—which causes peritoneal fluid absorption that cannot be easily quantified—cannot be fully excluded. Long-term follow-up data were available for only 40 of 124 horses. Finally, intraoperative fluid decisions were made by four different anaesthetists (one European diplomate), introducing potential variation in practice that reflects the real-world clinical setting. Fluid volumes are reported as total dose (mL/kg) rather than as a rate (mL/kg/h); as anaesthesia and surgical duration varied among horses, the same total dose may reflect different rates of administration, which could itself influence endothelial and glycocalyx effects. As fluid administration was determined by the attending clinician rather than randomised, horses receiving larger volumes may have differed systematically in disease severity (e.g., degree of shock, lactate, blood pressure), and although the association between fluid dose and TPP reduction persisted after adjustment for lesion type and intestinal tract involved in multivariable analysis, unmeasured severity indicators cannot be fully excluded as residual confounders. Clinical outcomes potentially related to hypoproteinaemia (POR, POI, hospitalisation length, survival) were not systematically analysed as primary endpoints of this study; a post hoc comparison of hospitalisation length by TPP group, and the structural exclusion of POI cases from the analytic cohort, are reported in Section 3.4 and discussed above, but should be interpreted with the appropriate caution given their post hoc nature. Mortality and postoperative reflux were not analysed in the present cohort, which by design excludes horses that died or received postoperative fluids; a dedicated analysis of perioperative risk factors for mortality and POR in the full, unselected hospital population is planned as a separate study. Finally, no outcome-based analysis (e.g., ROC curve) was performed to formally validate the proposed TPP ≥ 5 g/dL target, which should therefore be regarded as a pragmatic clinical target rather than an evidence-based threshold. 

## 5. Conclusions

Perioperative TPP reduction was common in horses undergoing colic surgery and was positively associated with cumulative crystalloid administration. This association remained statistically significant after adjustment for lesion type and intestinal tract involved; however, these variables capture only selected disease-related characteristics, and residual confounding by shock severity, lactate concentration, blood pressure, and other clinical determinants of fluid administration cannot be excluded. Separately, 52/124 horses (41.9%) reached a recovery TPP concentration ≤ 5.0 g/dL. 

Whether the observed TPP reductions and associated haemodilution adversely affect clinical outcomes cannot be determined from the present study. The association between recovery TPP ≤ 5.0 g/dL and hospitalisation length was identified post hoc, and the median difference was small (11 vs. 10 days). Potential relationships between haemodilution and intestinal oedema, endothelial glycocalyx injury, postoperative ileus, reflux, coagulation abnormalities, or survival remain biologically plausible but were not demonstrated by the present data.

Serial TPP monitoring provides a low-cost and immediately available measure of changes in plasma protein concentration and may be a useful adjunct to established clinical and haemodynamic monitoring during perioperative fluid therapy. However, the present study does not demonstrate that TPP can distinguish restoration of circulating volume from further fluid accumulation. The proposed ≥ 5.0 g/dL target should therefore be regarded as pragmatic and hypothesis-generating rather than as a validated treatment threshold. Prospective studies incorporating direct measures of circulating volume, cardiac output or fluid responsiveness and clinically relevant postoperative outcomes are required to determine whether TPP-guided fluid titration can improve perioperative fluid management or patient outcomes.

## Figures and Tables

**Figure 1:**
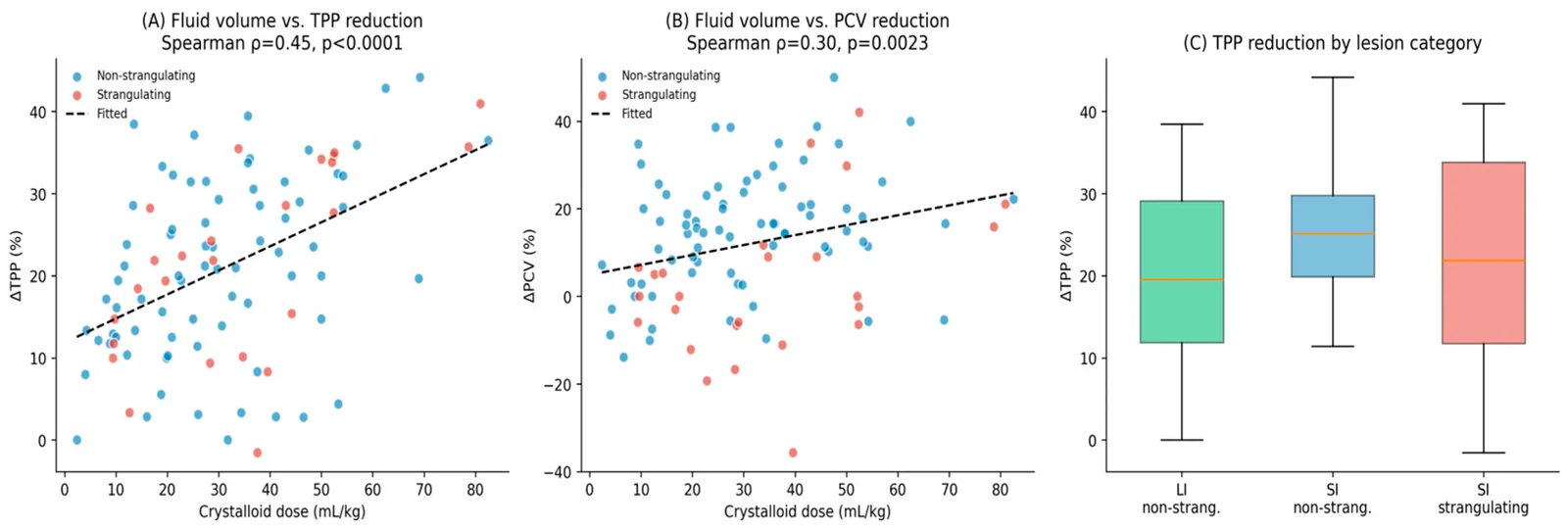
Relationship between perioperative crystalloid dose and haemodilution, and total plasma protein (TPP) reduction by lesion category. (**A**) Scatter plot of crystalloid dose (mL/kg) versus percentage reduction in TPP from admission to end of surgery (ΔTPP%), by lesion type (non-strangulating vs. strangulating), with fitted regression line (Spearman ρ = 0.45, *p* < 0.0001). (**B**) Scatter plot of crystalloid dose versus percentage reduction in packed cell volume (ΔPCV%), by lesion type, with fitted regression line (Spearman ρ = 0.30, *p* = 0.0023). (**C**) Boxplots of ΔTPP% by lesion category: large intestinal (LI) non-strangulating, small intestinal (SI) non-strangulating, and SI strangulating lesions. Horizontal lines within boxes indicate the median; box limits indicate the interquartile range.

**Table 1:** Type and amount of intravenous fluids administered perioperatively, stratified by group. LRS = Lactated Ringer’s solution; HES = hydroxyethyl starch 130/0.4; SI = small intestinal; LI = large intestinal; S = strangulating; NS = non-strangulating; TPP = total plasma protein; end-sx = end of surgery. * Data for SI subgroups derived from the companion study (Giusto et al.). Inter-group *p*-value for NS TPP ≤ 5 vs. NS TPP > 5: *p* = 0.156. Note: not all HES recipients were hypoproteinaemic; colloid administration was at the anaesthetist’s discretion, generally triggered by persistently low blood pressure, marked hypoproteinaemia, or clinical signs of inadequate perfusion despite crystalloid administration; a fully standardised protocol-driven trigger was not applied.

Group	*n*	Pathology Type	LRS mL/kg Median [Range]
All horses	124	SI + LI (all)	34.3 [9–99]
Group 1: TPP ≤ 5 g/dL at end-sx (SI subset) *	24	Strangulating + non-strangulating SI	31.2 [0–79.6]
Group 2: TPP > 5 g/dL at end-sx (SI subset) *	28	Strangulating + non-strangulating SI	24.0 [0–84.2]
Subgroup S TPP ≤ 5 *	13	Strangulating SI	35.3 [9.2–79.6]
Subgroup S TPP > 5 *	14	Strangulating SI	28.3 [8.8–61.2]
Subgroup NS TPP ≤ 5 *	11	Non-strangulating SI	56.8 [7.4–74.1]
Subgroup NS TPP > 5 *	14	Non-strangulating SI	31.5 [8.3–84.2]

**Table 2:** PCV and TPP values at arrival and end of surgery with percentage reductions, stratified by group (SI subgroup only). * Data from the companion study. PCV = packed cell volume; TPP = total plasma protein; Δ = median percentage reduction from arrival to end of surgery; end-sx = end of surgery.

Group	PCV Arrival %	PCV End-Sx %	TPP Arrival g/dL	TPP End-Sx g/dL	ΔPCV % Median	ΔTPP % Median
Group 1 (all SI, TPP ≤ 5) *	40 [25–72]	37 [26–60]	6.8 [5.0–8.5]	4.9 [3.6–5.0]	8.4	28.6
Group 2 (all SI, TPP > 5) *	40 [28–57]	37.5 [28–45]	7.0 [5.8–8.0]	5.8 [5.2–7.0]	6.7	16.1
Subgroup S TPP ≤ 5 *	34 [25–47]	34 [26–46]	6.4 [5.0–7.8]	5.0 [4.0–5.0]	0.0	24.2
Subgroup S TPP > 5 *	36 [28–50]	37.5 [29–45]	6.4 [5.8–8.0]	6.0 [5.1–7.0]	5.0	9.4
Subgroup NS TPP ≤ 5 *	45 [36–72]	38 [27–60]	7.0 [6.0–8.0]	5.0 [4.0–5.0]	16.7	37.5
Subgroup TPP > 5 *	42 [35–50]	36 [28–43]	7.2 [6.0–8.5]	6.0 [5.1–7.0]	17.5	20.0

**Table 3:** Total plasma protein (TPP, g/dL) values from arrival to POD 7 in 124 horses. Friedman test; superscript letters indicate statistically significant pairwise comparisons (all *p* < 0.0001). POD = postoperative day.

Parameter	Arrival	Recovery	POD 2	POD 5	POD 7
Median TPP (g/dL)	6.9 ^a,b^	5.4 ^a,c,d,e^	6.3 ^b,c^	6.6 ^d^	6.6 ^e^
Lower 95% CI	6.8	5.3	6.3	6.4	6.5
Upper 95% CI	7.0	5.5	6.5	6.6	6.7

**Table 4:** TPP (g/dL) from arrival to long-term follow-up (n = 40 horses). Friedman test; a: *p* = 0.016; b: *p* = 0.025.

Parameter	Arrival	Recovery	POD 2	POD 5	POD 7	Long-Term (≥2 mo)
Median TPP (g/dL)	7.0 ^a^	5.4 ^b^	6.4	6.4	6.4	6.4 ^a,b^
Lower 95% CI	6.6	5.1	6.2	6.2	6.2	6.1
Upper 95% CI	7.3	5.8	6.8	6.6	6.7	6.8

**Table 5:** PCV (%) from arrival to POD 7 in 124 horses. Friedman test; superscript letters: a,b,c,d: *p* < 0.0001; e: *p* < 0.04.

Parameter	Arrival	Recovery	POD 2	POD 5	POD 7
Median PCV (%)	38.5 ^a,b,c,d^	32.0 ^a^	33.0 ^b,e^	32.5 ^c^	32.0 ^d,e^
Lower 95% CI	38.2	31.8	32.6	31.7	31.5
Upper 95% CI	40.4	33.3	34.4	33.0	32.8

**Table 6:** PCV (%) from arrival to long-term follow-up. Friedman test; a: *p* = 0.018.

Parameter	Arrival	Recovery	POD 2	POD 5	POD 7	Long-Term (≥2 mo)
Median PCV (%)	38.0 ^a^	33.5	34.5	33.0	31.0	31.6 ^a^
Lower 95% CI	35.9	31.4	32.0	29.7	28.8	31.0
Upper 95% CI	40.8	34.5	36.6	33.6	33.1	35.5

## Data Availability

The raw data supporting the conclusions of this article will be made available by the authors on request.

## References

[1] Malbrain M.L.N.G., Langer T., Annane D., Gattinoni L., Elbers P., Hahn R.G., De Laet I., Minini A., Wong A., Ince C. (2020). Intravenous fluid therapy in the perioperative and critical care setting: Executive summary of the International Fluid Academy (IFA). Ann. Intensive Care.

[2] Muir W.W., Ueyama Y., Noel-Morgan J., Kilborne A., Page J. (2017). A systematic review of the quality of IV fluid therapy in veterinary medicine. Front. Vet. Sci..

[3] Czajka S., Marczenko K., Wlodarczyk M., Szczepańska A.J., Olakowski M., Mrowiec S., Krzych Ł.J. (2021). Fluid therapy in patients undergoing abdominal surgery: A bumpy road towards individualised management. Adv. Exp. Med. Biol..

[4] Myles P.S., Andrews S., Nicholson J., Lobo D.N., Mythen M. (2017). Contemporary approaches to perioperative IV fluid therapy. World J. Surg..

[5] Grass F., Lovely J.K., Crippa J., Hübner M., Mathis K.L., Larson D.W. (2020). Potential association between perioperative fluid management and occurrence of postoperative ileus. Dis. Colon Rectum.

[6] Giusto G., Vercelli C., Gandini M. (2021). Comparison of liberal and goal-directed fluid therapy after small intestinal surgery for strangulating lesions in horses. Vet. Rec..

[7] Archer D.C., Pinchbeck G.L., Proudman C.J. (2011). Factors associated with survival of epiploic foramen entrapment colic: A multicentre, international study. Equine Vet. J..

[8] Salem S.E., Proudman C.J., Archer D.C. (2016). Prevention of post operative complications following surgical treatment of equine colic: Current evidence. Equine Vet. J..

[9] Fielding L. (2014). Crystalloid and colloid therapy. Vet. Clin. N. Am. Equine Pract..

[10] Barton C., Hassel D.M. (2023). Critical Care of the Colic Patient: Monitoring, Fluid Therapy, and More. Vet. Clin. N. Am. Equine Pract..

[11] Muir W. (2023). Contemporary perspectives on perioperative fluid therapy. J. Am. Vet. Med. Assoc..

[12] Spier S.J., Meagher D.M. (1989). Perioperative medical care for equine abdominal surgery. Vet. Clin. N. Am. Equine Pract..

[13] Seahorn T.L., Cornick-Seahorn J. (1994). Fluid therapy. Vet. Clin. N. Am. Equine Pract..

[14] Crabtree N.E., Epstein K.L. (2021). Current concepts in fluid therapy in horses. Front. Vet. Sci..

[15] Muir W.W., Yiew X.T., Bateman S.W., Hahn R.G. (2026). Volume kinetics of lactated Ringer’s solution in adult horses. Equine Vet. J..

[16] Schoster A., Mitchell K., Auer J., Stick L., Kummerle J.M., Prange T. (2019). Fluids, electrolytes, and acid-base therapy. Equine Surgery.

[17] Corley K., Stephen J. (2008). The Equine Hospital Manual.

[18] Boscan P., Steffey E.P. (2007). Plasma colloid osmotic pressure and total protein in horses during colic surgery. Vet. Anaesth. Analg..

[19] Freeman D.E. (2018). Fifty years of colic surgery. Equine Vet. J..

[20] Schott H.C. (2003). Hydration, body fluid volumes, and fluid therapy—Are we moving forward as fast as we think?. J. Vet. Intern Med..

[21] Proudman C.J., Smith J.E., Edwards G.B., French N.P. (2002). Long-term survival of equine surgical colic cases. Part 1: Patterns of mortality and morbidity. Equine Vet. J..

[22] Smart L., Hughes D. (2021). The effects of resuscitative fluid therapy on the endothelial surface layer. Front. Vet. Sci..

[23] Hobbs K.J., Ludwig E.K., Martin E.M., Bayless R., Bauck A.G., Freeman D.E., Gonzalez L.M. (2025). Endothelial glycocalyx damage is associated with small intestinal disease in horses. Am. J. Vet. Res..

[24] Freeman D.E., Mooney A., Giguere S., Claire J., Evetts C., Diskant P. (2021). Effect of feed deprivation on daily water consumption in healthy horses. Equine Vet. J..

[25] Wendt-Hornickle E.L., Snyder L.B., Tang R., Johnson R.A. (2011). The effects of Lactated Ringer’s solution (LRS) or LRS and 6% hetastarch on the colloid osmotic pressure, total protein and osmolality in healthy horses under general anaesthesia. Vet. Anaesth. Analg..

[26] Breheny C.R., Perez-Accino Salgado J., Bommer N.X., Handel I., Gow A.G. (2019). Standard operating procedure reduces interoperator variation and improves accuracy when measuring packed cell volume. Vet. Rec..

[27] Proudman C.J., Edwards G.B., Barnes J., French N.R. (2005). Factors affecting long-term survival of horses recovering from surgery of the small intestine. Equine Vet. J..

[28] Boesch J.M. (2013). Anaesthesia for the horse with colic. Vet. Clin. N. Am. Equine Pract..

[29] Hanzawa K., Kubo K., Kai M., Hiraga A., Watanabe S. (1999). Effects of splenic erythrocytes and blood lactate levels on osmotic fragility of circulating red cells in thoroughbred horses during exercise. J. Equine Sci..

[30] Shah S.K., Moore-Olufemi S.D., Uray K.S., Jimenez F., Walker P.A., Xue H., Stewart R.H., Laine G.A., Cox C.S. (2010). A murine model for the study of oedema-induced intestinal contractile dysfunction. Neurogastroenterol. Motil..

